# Fluid Extracts

**Published:** 1869-04

**Authors:** John Cooper


### Fluid Extracts.

BY JOHN COOPER, M. D.

There is much being written of late in the various medical and pharmaceutical journals regarding the different varieties of the Fluid Extracts found in the market, but no one so far as I can find, has called attention to the mode of manufacturing them, in which lies the root of all the difficulty. I will endeavor to expose the various plans offered, and believe it possible on this ground to show that the process by which they are generally made, is so faulty and irrational as to explain why these most efficacious, convenient and elegant preparations are losing their hold on the profession.

A fluid extract in theory is a concentrated tincture, in one fluid ounce of which there is held in solution all the medicinal properties of one Troy ounce of crude drug; or in other words, all the therapeutical properties of 480 grains of crude drug are held in solution by 480 minims of fluid extract. Here is expressed the great value of these preparations, and if they are properly made, they should be efficient in a dose, the number of minims of which should equal the number of grains of crude drug indicated; but in practice, the profession has been disappointed in them, and they are universally given in doses larger than is required for the officinal tinctures, which are in amount of drug used in their preparation, nearly ten times weaker.

I have given of the fluid extract of ergot, as generally found in stores, 2 fluid ounces before getting the effect of 20 grains of the powdered drug. Here is a great discrepancy between the preparation as it should be, and as it actually is. Where is the fault? Surely not in the manufacturer, for as a general rule he is a high minded, honorable man, who would not intentionally give the physician an unreliable preparation, with which to fight off suffering and death.

Let us look at the various processes by which they are made, and see if a grave error has not been committed by the revisors of the Pharmacopœia, to which we can trace the difficulty.

A fluid extract is made only of those drugs whose active properties are included in the gums, resins, resenoids, volatile oils, and extractive matters. The first and last of these are soluble in water, and partially in alcohol; the rest are insoluble in water, and generally in alcohol. The gums are, as a rule, very inert as medicinal agents, and the point sought for is to get from the drug all of the others. It is desirable to have them all in the preparation, but no menstruum known will take them all up; so it is best to lose part of the gums, and secure the more active properties. To illustrate the officinal process, we will take buchu, as it is a simple extract, and made clearly by the general rule laid down for the pharmacist.

It is ordered to displace from 16 Troy ozs. of the leaves, in moderately fine p owder, 12 fluid ozs. of tincture, and set aside. So far good. Then pass through 32 fluid ozs. more of liquid, which is held to contain all the remaining active properties of the drug not in the first percolate. These added together would give 44 fluid ozs. of tincture, for 16 fluid ozs. of drug, which is wide of the point sought for.

To get this excess reduced to the proper strength, it is ordered to take this last percolate of 32 ozs. and place it in an open vessel over the water bath and evaporator, by a heat not exceeding 150 deg., to 4 fluid ozs., the amount needed to make up the 16 fluid ozs. This is the great error, and it is fatal to the preparation. This last percolate is alcohol of 0.835 sp. gr. holding in *solution* all the resins, resinoids, volatile oils, and extractive matters *not taken up by the first percolate.* These are insoluble in water. The prolonged heat applied to the vessel to reduce the solution most certainly drives off first the alcohol and the volatile oils, leaving the water, the alcohol and oils being the most volatile. As there is nearly 20 per cent. of water in the menstruum, we have quite 6 oz. (so that the 4 oz.) of it in the 32 ozs. cannot retain one particle of alcohol, and of course the active properties as shown above not being soluble in water, are deposited. This leaves these 4 ozs. worthless, and the active properties of the 32 ozs. destroyed, so far as the final preparation is concerned. Instead of throwing this worthless result upon the ground, it is ordered to mix it with the first percolate, so as to gain the quantity desired, at a reckless loss in quality; for the 12 ozs. like the 32 ozs. holds in solution only those active properties soluble in alcohol of the sp. gr. 0.835, and to reduce it by the addition of 4 ozs. of water, causes a further deposit just as certainly as 4 ozs. of water added to 12 ozs. of saturated tincture of camphor will deposit the gum. Then it is wisely ordered to set it aside for 24 hours, to allow the precipitate to deposit fully, and then filter through paper to get a clear preparation, which will look nicely if it is almost deficient in strength.

This is the officinal process as laid down in the last edition of the Pharmacopœia, and can any one wonder why the fluid extracts are losing the proud position they occupied at first?

To obviate these faults, some manufacturers are now making them in vacuo, but they have overlooked the fact that the fault lies not only in the contact with the air necessary by the officinal mode, but also as in the loss of alcohol, and it is evident to any one who will think of it, that the air pump will exhaust the tincture in the pan, of its alcohol first, and leave the preparation in a worse condition than does the officinal plan, and in practice they as a general rule are poorer in therapeutical properties than the officinal tinctures.

To fully meet all the objections offered to these modes, I know of none so rational and complete as the process of N. Spencer Thomas, which is now adopted by Messrs. Iredell & Co., Philadelphia. They take 100 pounds of drug ground properly, and macerate in 25 pounds of menstruum of proper strength for 24 hours in an air-tight vessel, then subject the mass to a pressure of over 800 pounds to the square inch of presenting surface, which squeezes from the drug the menstruum saturated with the medicinal properties; this is carefully excluded from the air to prevent loss of alcohol, and set aside. The mass is again macerated as before and pressed, and the liquid added to the first pressing. The same process is repeated again, and the result of the three pressings is mixed and weighed, and the amount necessary for the 100 pounds required, is put upon the mass and after 24 hours subjected to the last pressing. This liquid is mixed with that from the three former pressings, and the result is just 100 pounds of fluid extract, one minim of which will most fully represent one grain of the drug. Here, is no loss of alcohol, no heat, no evaporation, nor prolonged contact with the air, and the preparation is a concentrated liquid holding in solution all the medicinal virtues of the drug as formed by nature.

